# Navigating the gastric frontier of mucosal immunity against *Helicobacter pylori*

**DOI:** 10.3389/fimmu.2026.1727910

**Published:** 2026-07-28

**Authors:** Yan-Ruide Li, Yuning Chen, Yichen Zhu, Xinyuan Shen, Lili Yang

**Affiliations:** 1 Department of Microbiology, Immunology & Molecular Genetics, University of California, Los Angeles, Los Angeles, CA, United States; 2 Department of Bioengineering, University of California, Los Angeles, Los Angeles, CA, United States; 3 Microbiome Vaccine Program, Goodman-Luskin Microbiome Center, University of California, Los Angeles, Los Angeles, CA, United States; 4 Molecular Biology Institute, University of California, Los Angeles, Los Angeles, CA, United States; 5 Eli and Edythe Broad Center of Regenerative Medicine and Stem Cell Research, University of California, Los Angeles, Los Angeles, CA, United States; 6 Jonsson Comprehensive Cancer Center, David Geffen School of Medicine, University of California, Los Angeles, Los Angeles, CA, United States; 7 Parker Institute for Cancer Immunotherapy, University of California, Los Angeles, Los Angeles, CA, United States

**Keywords:** cell therapy, gastric adenocarcinoma, Helicobacter pylori, immune evasion, mucosa-associated invariant T (MAIT) cell, mucosal immunity, ulcer

## Abstract

*Helicobacter pylori (H. pylori)* is a globally prevalent bacterium that causes chronic infection and, while often asymptomatic, is a major cause of gastritis, peptic ulcer disease, and a risk factor for gastric adenocarcinoma and mucosa-associated lymphoid tissue (MALT) lymphoma. Infection triggers robust innate and adaptive immune responses in the gastric mucosa, including immune cell recruitment and pro-inflammatory cytokine production; however, the bacterium frequently persists by evading host immunity through mechanisms such as CagA-mediated epithelial damage and modulation of heat shock proteins. This review examines the interplay between mucosal immunity and *H. pylori*, with a particular focus on the unique functional roles of mucosa-associated invariant T (MAIT) cells in orchestrating host defense. We also explore therapeutic strategies that leverage mucosal immunity to combat *H. pylori*, including immune-based interventions to eradicate infection and approaches targeting *H. pylori*-associated diseases, such as gastric cancer, autoimmune disorders, and peptic ulcer disease.

## Introduction

*Helicobacter pylori* (*H. pylori*) remains one of the most prevalent bacterial pathogens worldwide, causing chronic infection in humans, with a global adult prevalence of infection estimated at 43.9% between 2015 and 2022 (1, 2). Infection is typically acquired during childhood, and although most cases are asymptomatic, *H. pylori* is a major cause of chronic gastritis and peptic ulcer disease and is a significant risk factor for gastric adenocarcinoma and mucosa-associated lymphoid tissue (MALT) lymphoma (3–6). Risk factors for infection include lower socioeconomic status, poor sanitation, and certain ethnic backgrounds. Successful eradication of *H. pylori* has been shown to resolve chronic gastritis and peptic ulcers, reduce the risk of ulcer bleeding in aspirin users, and significantly lower the incidence of gastric cancer, highlighting the clinical importance of effective management (7–9).

Beyond its clinical manifestations, *H. pylori* exerts profound effects on gastric mucosal immunity. Infection triggers robust innate and adaptive immune responses, including recruitment of immune cells and secretion of pro-inflammatory cytokines. Despite these responses, the bacterium often persists, resulting in chronic inflammation that damages the gastric mucosa and increases the risk of ulcers, gastric adenocarcinoma, and lymphoma (10–14). Persistent infection underscores the bacterium’s ability to evade immune surveillance, establishing a long-term niche in the host stomach.

*H. pylori* has evolved multiple virulence mechanisms that enable persistent colonization of the harsh gastric environment and contribute to disease pathogenesis. Survival in the acidic stomach is primarily mediated by urease, which hydrolyzes urea to generate ammonia and carbon dioxide, creating a more favorable local microenvironment (10–14). Flagella-driven motility and chemotaxis facilitate penetration of the gastric mucus layer, while adhesins, including blood group antigen-binding adhesin (BabA) and sialic acid-binding adhesin (SabA), promote stable attachment to gastric epithelial cells. In addition, *H. pylori* produces enzymes with mucinase activity that degrade gastric mucus glycoproteins, facilitating bacterial penetration through the mucus layer and enhancing access to the epithelial surface. This process promotes stable colonization and contributes to persistent infection by enabling close interactions between the bacterium and the gastric epithelium (10–14). Major virulence factors such as cytotoxin-associated gene A (CagA) and vacuolating cytotoxin A (VacA) further promote epithelial injury, disrupt host signaling pathways, and facilitate long-term bacterial persistence. These virulence determinants establish the foundation for chronic colonization and shape subsequent interactions between *H. pylori* and the host immune system.

The interaction between *H. pylori* and the host immune system involves complex crosstalk between epithelial cells, innate immune cells such as macrophages and dendritic cells (DCs), and adaptive immune components including T and B lymphocytes (10, 15–17). Bacterial components, such as lipopolysaccharide (LPS), activate epithelial Toll-like receptor 4 (TLR4), initiating innate immune signaling (18, 19). The resulting recruitment of immune cells and production of pro-inflammatory cytokines, including interleukin-1 (IL-1), IL-6, and IL-8, amplifies inflammation and contributes to tissue remodeling. Adaptive responses lead to the formation of lymphoid follicles and generate both humoral and cell-mediated immunity, yet these mechanisms are frequently subverted by the bacterium, preventing clearance (17, 20, 21).

*H. pylori* employs multiple immune evasion strategies that facilitate its persistence (16, 22, 23). The bacterium can directly damage gastric epithelial cells through type IV secretion systems that inject effectors such as CagA, inducing apoptosis and compromising the gastric barrier. In addition, *H. pylori* suppresses host immunity through various mechanisms, including downregulation of heat shock proteins (HSPs), which modulate immune responses (16, 22, 23). These evasion strategies allow chronic colonization and sustained inflammation, providing a mechanistic basis for *H. pylori*-associated pathologies and emphasizing the importance of understanding mucosal immune responses for the development of effective therapies.

In this review, we examine the complex interplay between mucosal immunity and *H. pylori*, with a particular focus on MAIT cells, highlighting their unique functional properties and dual Th1/Th17 responses (24–26). We discuss the balance between Th1, Th17, and regulatory T (Treg) cells in shaping the gastric immune environment and influencing bacterial persistence or clearance. In addition, we provide a critical overview of therapeutic strategies, including antibiotic treatment and the challenges posed by resistance, immunomodulatory approaches, and vaccine development targeting mucosal immunity, emphasizing emerging MAIT cell-based interventions. Finally, we explore cutting-edge technologies such as single-cell RNA sequencing and spatial transcriptomics, consider the implications of immune heterogeneity for personalized approaches, and outline current gaps in knowledge and key research questions that could guide future investigations.

## Mucosal immune responses in *H. pylori* infection

The gastric epithelium constitutes a specialized mucosal barrier that provides the first line of defense against ingested pathogens including *H. pylori*. Comprised of a single layer of epithelial cells, it preserves tissue integrity and regulates communication between the gastric lumen and underlying immune compartments (27, 28). This barrier is reinforced by a protective mucus layer, which acts as both a physical and biochemical shield by trapping microbes and facilitating their clearance. In addition, epithelial cells secrete antimicrobial peptides, including defensins and cathelicidins, which exert direct bactericidal activity and modulate local immune responses (29). *H. pylori*, however, has evolved strategies to interact intimately with this barrier, adhering to epithelial cells and translocating virulence factors such as CagA via a type IV secretion system. These interactions compromise epithelial integrity, perturb signaling pathways, and promote pro-inflammatory responses. Through pattern recognition receptors, gastric epithelial cells sense bacterial components such as LPSs and flagellin, initiating innate immune responses that recruit and activate immune cells, thereby forming a critical interface between host defense and bacterial persistence.

Once the gastric mucosal barrier is breached, *H. pylori* induces robust innate immune responses that shape the course of infection. Recognition of bacterial ligands by pattern recognition receptors, including TLRs and NOD-like receptors (NLRs), activates NF-κB signaling and drives the production of pro-inflammatory cytokines (e.g., IL-1β, IL-6, IL-8, TNF-α) and chemokines (e.g., IL-8), which orchestrate the recruitment of neutrophils and monocytes. While neutrophils contribute to bacterial control, their release of reactive oxygen species and proteolytic enzymes also inflicts collateral tissue damage (15, 30). Macrophages and DCs function as key phagocytes and antigen-presenting cells, producing inflammatory mediators, but *H. pylori* can manipulate their activation states, for example by skewing macrophages toward anti-inflammatory phenotypes to promote persistence. Innate lymphoid cells, such as NK cells and gamma-delta (γδ) T, are also activated and contribute to early IFN-γ and IL-17 production (17). Despite these multifaceted innate defenses, *H. pylori* frequently establishes chronic infection by subverting innate immune pathways, underscoring its ability to exploit host immunity for long-term colonization.

Adaptive immunity further shapes the host–pathogen interaction but rarely achieves sterilizing clearance of *H. pylori*. Infection is characterized by activation of CD4^+^ T helper subsets, particularly Th1 and Th17 cells, which produce IFN-γ and IL-17 to drive macrophage activation, neutrophil recruitment, and sustained mucosal inflammation (31). In parallel, Tregs are expanded, secreting IL-10 and TGF-β to suppress effector responses, thereby facilitating immune tolerance and bacterial persistence (32). The balance among Th1, Th17, and Treg responses critically determines whether host immunity favors protection or progression to chronic gastritis and gastric cancer. The persistence of *H. pylori* despite vigorous adaptive immune activation highlights the pathogen’s sophisticated immune evasion strategies and points to potential therapeutic opportunities that target T cell regulation and function.

## Human MAIT cells and *H. pylori* infection

MAIT cells have recently emerged as critical players at the interface of innate and adaptive immunity, particularly within mucosal tissues (24, 33, 34). These cells recognize riboflavin-derived microbial metabolites presented by the non-polymorphic MR1 molecule and exert both Th1- and Th17-like effector functions. Through the production of cytokines such as IFN-γ, TNF-α, and IL-17A, MAIT cells contribute to antimicrobial defense while also amplifying local inflammatory responses (33, 35–38).

Enriched at mucosal barriers, MAIT cells serve as rapid responders against bacterial and viral infections. In addition to direct antimicrobial activity, they promote the recruitment of other immune populations and can enhance B cell responses, thereby supporting humoral immunity and vaccine efficacy (39–42). Beyond host defense, MAIT cells also play roles in maintaining tissue homeostasis and promoting wound repair. However, their potent effector functions can contribute to immunopathology under dysregulated conditions (43–46). Thus, MAIT cells represent a double-edged sword in immunity, participating in both protective and pathogenic processes across a spectrum of diseases.

**Figure 1:**
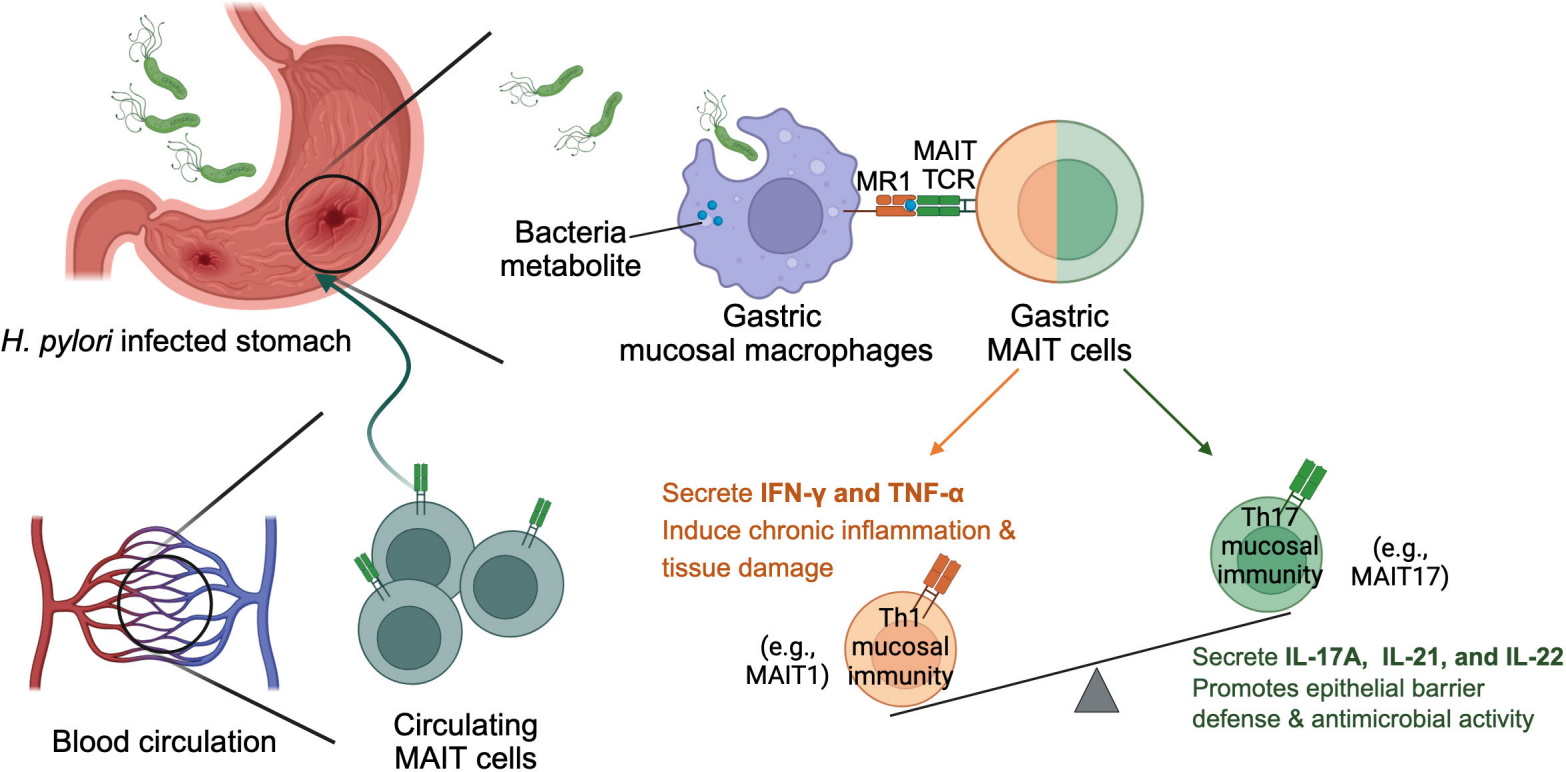
Schematic illustration of the dual protective and pathogenic roles of MAIT cells in chronic *H. pylori* infection. Gastric macrophages present riboflavin metabolites via MR1 to tissue-resident MAIT cells. Upon activation, MAIT cells polarize toward Th1-like (MAIT1) responses, producing IFN-γ and TNF-α that drive inflammation and tissue damage, or Th17-like (MAIT17) responses, producing IL-17A, IL-21, and IL-22 that promote epithelial defense and antimicrobial activity. Circulating MAIT cells are reduced in peripheral blood, whereas gastric tissue–resident MAIT cells are enriched, underscoring their compartmentalized role in disease progression.

MAIT cells play a pivotal role in the host immune response to *H. pylori* infection, exhibiting both protective and pathogenic functions. *H. pylori* possesses the riboflavin synthesis pathway, enabling the production of ligands recognized by the MAIT TCR. Upon activation, MAIT cells secrete pro-inflammatory cytokines such as IFN-γ and TNF-α, promoting local inflammation characteristic of chronic gastritis and facilitating recruitment of additional immune effectors (47) (**Figure 1**).

Gastric MAIT cells exhibit a tissue-resident phenotype, marked by expression of CD69 and CD103, indicating their readiness to respond rapidly to local pathogens (47). Functional studies demonstrate that both gastric and circulating MAIT cells can recognize *H. pylori*. Specifically, CD8^+^ and CD4^−^CD8^−^ (double-negative) MAIT subsets respond to *H. pylori*-infected macrophages in an MR1-restricted manner by producing cytokines (IFN-γ, TNF-α, IL-17A) and displaying cytotoxic activity (47). Notably, MAIT cell frequencies differ between systemic and mucosal compartments: blood MAIT cell counts are significantly reduced in *H. pylori*-positive individuals compared to uninfected controls, whereas gastric MAIT cell frequencies remain relatively unchanged. Furthermore, the majority of gastric MAIT cells (>80%) express tissue-resident markers, in contrast to peripheral blood MAIT cells (<3%), highlighting their strategic localization for rapid mucosal immune responses (47). The reduction in circulating MAIT cells may reflect their preferential recruitment from the peripheral blood to inflamed gastric mucosa during *H. pylori* infection and/or activation-induced changes in their frequency. Although the precise developmental and migratory relationship between circulating and tissue-resident MAIT cells remains incompletely understood, current evidence suggests that these populations exhibit compartment-specific phenotypes and functions that together contribute to mucosal immune surveillance and host defense.

Another study showes that in *H. pylori*-associated gastritis, the OX40/OX40L signaling axis, a costimulatory pathway involving the T-cell receptor OX40 (CD134) and its ligand OX40L (CD252), has emerged as a critical regulator of MAIT cell-mediated mucosal immunity (48). Elevated expression of OX40 on mucosal MAIT cells, together with increased OX40L levels on DCs in infected patients compared with healthy controls, suggests enhanced co-stimulatory interactions within the gastric microenvironment (48). Activation of this pathway drives MAIT cell proliferation and induces substantial IL-9 production, promoting a highly activated phenotype. Notably, IL-9 secretion by MAIT cells correlates positively with markers of gastric inflammation, implicating IL-9–producing MAIT cells as contributors to disease pathology (48). These observations position the OX40/OX40L axis as a central mediator of MAIT cell–driven inflammation and highlight its potential as a therapeutic target in *H. pylori*-induced gastritis.

Collectively, these findings underscore the compartmentalized nature of MAIT cell responses during *H. pylori* infection, revealing a dichotomy between systemic and mucosal immunity (**Figure 1**). They provide important insights into the functional specialization of MAIT cells in peripheral versus tissue-resident compartments and their dual role in mediating host defense and contributing to chronic gastric inflammation.

## Therapeutic insights and vaccination strategies targeting mucosal immunity

### Human MAIT cell-based immunotherapy

Given their unique biology, tissue residency, and responsiveness to riboflavin-derived antigens, MAIT cells are increasingly being explored as novel candidates for immunotherapy. Unlike conventional T cells, MAIT cells are restricted by the highly conserved MR1 molecule, which is nearly monomorphic across the human population. This feature provides a strong rationale for the development of “universal” MAIT cell–based therapies that can overcome the limitations of HLA-restriction and interindividual variability inherent in conventional T cell approaches (33, 49–51).

One promising strategy for harnessing MAIT cells in immunotherapy involves the use of synthetic antigen delivery systems to selectively activate these cells *in vivo*. The potent MR1-binding ligand, 5-(2-oxopropylideneamino)-6-D-ribitylaminouracil (5-OP-RU), is a riboflavin metabolite derivative capable of inducing strong TCR-mediated MAIT cell activation (52–54). Formulating 5-OP-RU within lipid nanoparticles (LNPs) enables efficient trafficking to antigen-presenting cells (APCs), protects the unstable ligand from rapid degradation, and allows spatiotemporally controlled release. Preclinical studies have demonstrated that 5-OP-RU drives robust MAIT cell expansion and effector activation, leading to heightened cytokine production (e.g., IFN-γ, TNF-α, IL-17) and improved antimicrobial and antitumor responses (25, 54, 55). Slow-release formulations may further enhance local bioavailability and sustain MAIT engagement at mucosal or tumor sites. This approach provides a pharmacological means of modulating MAIT responses *in situ*, either as monotherapy or in combination with cancer vaccines, immune checkpoint inhibitors, or conventional therapies to augment efficacy.

**Figure 2:**
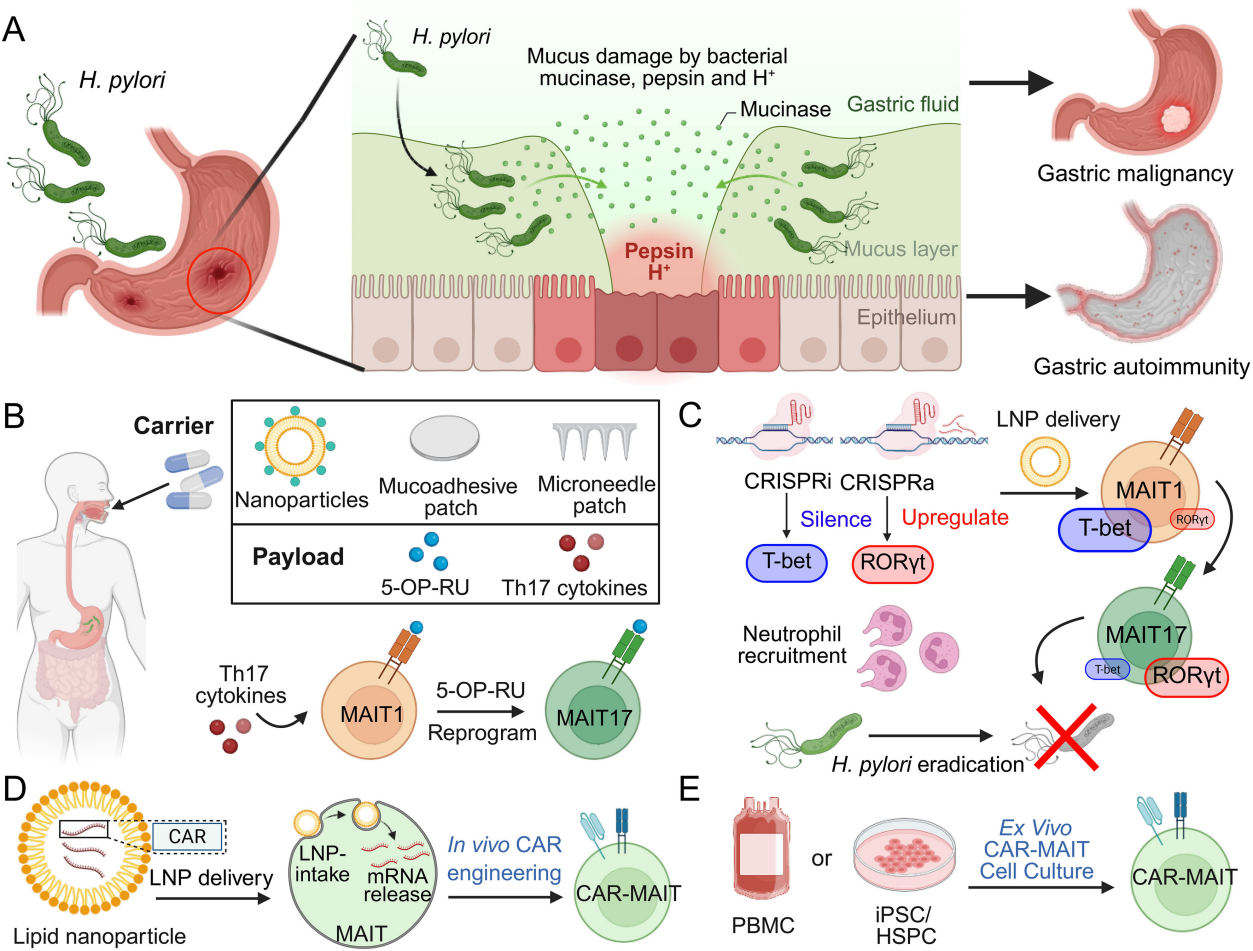
Strategies to reprogram and engineer MAIT cells for immunotherapy against *H. pylori* infection and its associated gastric diseases. **(A)** Chronic *H. pylori* colonization–induced mucus layer degradation by bacterial mucinase, pepsin, and gastric acid leads to epithelial injury and predisposing to gastric malignancy and autoimmunity. **(B)** Local delivery of 5-OP-RU and Th17-polarizing cytokines via nanoparticles, mucoadhesive patches, or microneedle patches to reprogram MAIT1 cells toward MAIT17 responses that enhance epithelial defense and antimicrobial activity. **(C)** LNP-mediated CRISPR transcriptional modulation silencing T-bet and upregulating RORγt in order to drive MAIT17 polarization, neutrophil recruitment, and enhanced *H. pylori* clearance. **(D)** *In vivo* chimeric antigen receptor (CAR) engineering of endogenous MAIT cells via lipid nanoparticle (LNP) delivery of CAR constructs, enabling direct targeting of *H. pylori*–driven pathology. **(E)** *Ex vivo* CAR-MAIT engineering from peripheral blood mononuclear cell (PBMC)-, hematopoietic stem and progenitor cell (HSPC)-, or induced pluripotent stem cell (iPSC)-derived MAIT cells, allowing expansion, genetic modification, and adoptive transfer as an off-the-shelf therapeutic product.

Beyond systemic administration, localized delivery platforms may further enhance MAIT cell-based immunotherapy by increasing antigen availability within the gastric mucosa while minimizing systemic immune activation. Nanoparticles, mucoadhesive formulations, and microneedle-based delivery systems have the potential to provide sustained local release of MR1 ligands, such as 5-OP-RU, together with immunomodulatory cytokines that promote MAIT17 polarization (**Figure 2**). Reprogramming MAIT cells toward a MAIT17-like phenotype may strengthen epithelial barrier function, enhance antimicrobial peptide production, and promote neutrophil recruitment, thereby improving mucosal protection against *H. pylori* (**Figure 2**). Nevertheless, these approaches remain largely preclinical, and important challenges, including efficient gastric delivery, maintenance of phenotypic stability, potential off-target immune activation, and the balance between protective immunity and excessive inflammation, must be addressed before clinical translation.

In addition, genetic engineering of MAIT cells represents an emerging frontier. Advances in *in vivo* gene-editing technologies—including LNP-delivered CRISPR/Cas9 systems and synthetic mRNA constructs—offer opportunities to enhance T cell functional potency, prolong persistence, or redirect specificity through chimeric antigen receptor (CAR) expression (56, 57). Given their preferential homing to mucosal tissues, rapid effector responses, and intrinsic ability to secrete both Th1- and Th17-type cytokines, engineered MAIT cells are particularly well-suited for targeting solid tumors within mucosal environments, such as gastric, colorectal, and hepatobiliary cancers (26, 58–60). Together, pharmacological and genetic strategies highlight the potential of MAIT cells as a novel and versatile platform for next-generation cancer immunotherapy.

The development of off-the-shelf MAIT and CAR-engineered MAIT (CAR-MAIT) cell therapies represents a particularly promising avenue for translational immunotherapy (58, 61–64). MAIT cells can be efficiently expanded *ex vivo* and genetically engineered from healthy donor sources or pluripotent stem cells to generate robust and standardized cellular products (56, 59, 65, 66). These cells could be harnessed to treat a wide spectrum of conditions, including cancers, autoimmune disorders, chronic ulcers, and, notably, *H. pylori*–associated diseases. For example, *H. pylori*–driven gastric cancer may represent an ideal target for adoptive MAIT cell therapy, in which unmodified MAIT cells exploit their innate antimicrobial and cytotoxic properties, or CAR-engineered MAIT cells are redirected toward tumor-associated antigens to enhance therapeutic efficacy. A key advantage of MAIT cell–based therapies is their restriction to the non-polymorphic MR1 molecule, which circumvents the challenges of HLA mismatch and minimizes the risk of graft-versus-host disease (GvHD) (65, 67, 68). This feature enables the generation of universally compatible, allogeneic “off-the-shelf” products, eliminating the need for patient-specific manufacturing and thereby facilitating rapid and scalable clinical deployment. Furthermore, MAIT cells’ inherent tissue-homing capacity, particularly to mucosal sites, coupled with their ability to secrete Th1- and Th17-associated cytokines and execute cytotoxic functions, positions them as uniquely suited to target diseases of the gastrointestinal tract. In *H. pylori*–induced pathology, this could include chronic gastritis, peptic ulcers, and gastric carcinoma, where both protective and pathogenic roles of MAIT cells have been implicated. Importantly, CAR-engineered MAIT cells could expand the therapeutic repertoire beyond infection-associated cancers to include solid tumors that are traditionally resistant to CAR-T cell therapies, leveraging MAIT cells’ tissue residency and rapid effector response (25, 69). Their compatibility with established cell engineering pipelines further underscores the feasibility of developing next-generation universal CAR-MAIT cell platforms. Collectively, these attributes suggest that off-the-shelf MAIT and CAR-MAIT cell therapies hold substantial promise as innovative immunotherapeutic strategies, with potential to complement or even surpass existing CAR-T and CAR-NKT platforms in *H. pylori*–related diseases and other difficult-to-treat malignancies.

Collectively, these strategies—including antigen-specific activation using 5-OP-RU delivered via LNPs, *in vivo* genetic engineering to enhance effector functions, and the generation of universal off-the-shelf MAIT or CAR-MAIT cell products—underscore the translational potential of MAIT cells as next-generation cellular immunotherapies for the treatment of *H. pylori* infection and associated pathologies (**Figure 2**). Such approaches could be particularly impactful in *H. pylori*–driven diseases, including chronic gastritis, peptic ulcer disease, and gastric cancer, where MAIT cells are known to play dual roles in both host protection and immunopathology.

### Antibiotic treatment and challenges with resistance

Eradication therapy for *H. pylori* has shifted decisively toward regimens that overcome widespread antimicrobial resistance and ensure potent acid suppression. The 2024 American College of Gastroenterology (ACG) guideline recommends 14-day optimized bismuth quadruple therapy (BQT) as the preferred empiric option for treatment-naïve patients when susceptibility is unknown; clarithromycin-based triple therapy should be avoided unless macrolide susceptibility has been documented (70, 71). Similar caution applies to levofloxacin-containing regimens (72). The guideline also endorses vonoprazan-based options and rifabutin triple therapy as reasonable alternatives, with universal test-of-cure required after treatment. European consensus aligns with this direction. The Maastricht VI/Florence report emphasizes susceptibility-guided therapy, advising against clarithromycin triple therapy in regions where clarithromycin resistance exceeds 15% and highlighting the role of molecular testing to inform first-line and rescue choices (73).

Rising primary resistance underpins these recommendations. Although situation varies by region, a 2024 global meta-analysis reports substantial primary resistance to commonly used agents, such as clarithromycin, metronidazole, levofloxacin, reinforcing the need to tailor therapy to local epidemiology and prior antibiotic exposure (74). Real-world registry data from Europe show that adherence to guideline-concordant single-capsule bismuth-quadruple therapy for 10 days or concomitant treatment with clarithromycin-amoxicillin-metronidazole/tinidazole for 14 days improves effectiveness, with overall eradication rates increasing as practice moved away from shorter, triple-therapy protocols (75, 76). Potassium-competitive acid blocker (PCAB) regimens further address acid suppression-related failures. In the United States, vonoprazan-amoxicillin dual therapy and vonoprazan-amoxicillin-clarithromycin triple therapy received FDA approval based on randomized trials, offering higher intragastric pH and once- or twice-daily dosing frameworks; however, clarithromycin-containing packages should still be reserved for susceptible infections (77–80).

One challenge is rapid, noninvasive susceptibility testing. While culture remains the reference, stool-based molecular assays that detect 23S rRNA mutations, such as A2143G, A2142G, and A2142C, which are predictive of clarithromycin resistance can expedite tailored therapy and are increasingly available in clinical laboratories. Consensus guidance notes expanding roles for such molecular tools, particularly where culture capacity is limited (73, 81). Moreover, the ACG calls for a test of cure by urea breath test, stool antigen, or biopsy at least 4 weeks after antibiotics, withholding proton pump inhibitor (PPI)/PCAB therapy for about 2 weeks beforehand to avoid false negatives (79).

### Immunomodulatory therapies

Immunomodulatory therapies refer to interventions that modify the host immune response or the gastric mucosal microenvironment to enhance bacterial clearance, reduce inflammation, or improve treatment outcomes. Although the approaches discussed in this section act through distinct biological mechanisms, they share the common goal of complementing conventional antibiotic therapy by strengthening host defense or promoting mucosal homeostasis. Accordingly, probiotics primarily modulate the gut microbiota and mucosal immune responses, lactoferrin exerts antimicrobial and immunoregulatory effects, vitamin D enhances innate immunity through induction of antimicrobial peptides, and rebamipide promotes mucosal protection while attenuating inflammatory signaling.

Beyond traditional antibiotic treatment, there is growing interest in immunomodulatory approaches that can boost eradication efficacy, blunt antibiotic-related adverse events that undermine adherence, and mitigate collateral microbiome effects (73, 79, 82, 83). Current adult guidelines are cautious: the 2024 ACG guideline concludes there is insufficient evidence to recommend routine probiotics to improve efficacy or tolerability, whereas the Maastricht VI/Florence consensus report acknowledges that certain probiotic preparations reduce GI side effects and may confer a modest benefit on eradication, while also flagging concerns about selecting resistance within the commensal resistome (73, 79). These statements frame a pragmatic, individualized use of immunomodulators alongside susceptibility-guided antibiotics and mandatory test-of-cure.

Several findings have indicated the feasibility of immunomodulatory therapies. For example, multiple recent meta-analyses report that adding probiotics to standard therapy is associated with higher eradication rates and fewer adverse events, though effect sizes vary by strain, dose, and timing (83, 84). Meta-analyses have demonstrated that several bacteria species, including *Lactobacillus* spp. and *Saccharomyces boulardii*, primarily reduce treatment-related adverse events and improve treatment tolerability rather than exert direct anti-*H. pylori* activity. In addition to probiotics, the iron-binding glycoprotein lactoferrin has antimicrobial and anti-inflammatory activity in the gastric niche, and adjunct trials suggest improved eradication of *H. pylori* and tolerability when added to legacy triple or sequential regimens, although findings are mixed and larger contemporary randomized clinical trials (RCTs) are needed (85, 86).

Another way to reduce gastric *H. pylori* burden is to induce host-defense peptide, which can be achieved by upregulating vitamin D3 receptor (VDR) and antimicrobial peptide (CAMP/LL-37) (87, 88). A clinical trial has tested the combinatorial therapy of oral vitamin D3 administration with conventional *H. pylori* triple therapy with pending results (NCT03142620). Therapeutic strategies have focused on mucosal protection as well. The mucosal protectant rebamipide, which induces gastric prostaglandins synthesis and reduces NF-κB/IL-8 signaling, has shown higher eradication rates for *H. pylori* when added to dual-therapy backbones in meta-analyses, with less consistent benefits when layered onto triple therapy (89, 90).

### Vaccine development targeting mucosal immunity

No vaccine against *H. pylori* is currently licensed, but mucosal vaccines remain a high-priority strategy to control infection, reduce antibiotic use, and blunt resistance pressure (91). One phase III, randomized, double-blind, placebo-controlled trial testing a three-dose oral recombinant *H. pylori* vaccine in *H. pylori*-naïve children showed 71.8% protection at the first year and waning to 55.8% by the third year. This vaccine contains a fusion of urease B subunit and *E. coli* heat-labile enterotoxin B subunit (LTB), proved to be safe and immunogenic (92, 93). Follow-up reviews continue to cite this study as proof-of-concept for pediatric prophylaxis, while noting the absence of comparable adult efficacy data (94).

Since *H. pylori* colonizes the gastric mucosa, vaccine antigens are commonly delivered with mucosal adjuvants to elicit strong local and systemic immunity, including cholera toxin B subunit (CTB), LTB, and *E. coli* double-mutant heat-labile toxin (dmLT) to drive robust mucosal and systemic responses (95, 96). Beyond classical adjuvants, the neutrophil-activating protein (HP-NAP), a miniferritin produced by *H. pylori*, is being explored as both antigen and immunostimulant to enhance Th1 responses in vaccine constructs (97, 98).

Antigen choices span urease (UreA/UreB), virulence factors (CagA, VacA), adhesins (HpaA), and multi-epitope cocktails; platforms include purified proteins, DNA, viral or bacterial vectors (e.g., *Lactococcus/Lactobacillus*), and outer-membrane vesicles (99–102). Across candidates, prophylactic vaccination before exposure appears more promising than therapeutic vaccination of already infected adults, likely due to gastric immune tolerance and immune-evasion mechanisms that impede sterilizing clearance (103–105).

Key obstacles remain. Protection waned over time in the pediatric oral vaccine, and strain diversity plus immune modulation by *H. pylori* challenge long-term coverage, highlighting the importance of maintaining durability and breadth (91, 93). Moreover, while dmLT and related mucosal adjuvants enhance responses, optimal dosing and reactogenicity profiles must be defined for large-scale pediatric programs (106). Following administration, prophylactic trials require prolonged follow-up to monitor adverse effects and ensure safety. These constraints indicate that progress will hinge on achieving broad, durable protection within dosing and safety parameters suitable for pediatric deployment.

### Prospects for combination strategies

Existing data suggest that BQT performs similarly to non-bismuth concomitant therapy (70, 107), with some analyses showing a modest advantage for BQT, likely because bismuth adds activity in resistant infections (108, 109). Preclinical studies have indicated bismuth salts impair *H. pylori* metal homeostasis and can enhance activity against resistant strains, supporting its role as a resistance-buffering co-agent (110, 111). PCAB-based pairings are reshaping combinations by trading breadth for depth (77, 112). Vonoprazan-amoxicillin dual therapy (VA) delivers strong acid control with a single antibiotic, offering advantages and guideline-listed use as a 14-day empiric alternative in appropriate patients (78, 107). Some clinical data support that VA works comparable to PCAB, though effectiveness appears region- and regimen-dependent (113, 114).

Two additional strategies can strengthen combination strategies. First, susceptibility-guided therapy (SGT) via culture or noninvasive stool PCR for 23S rRNA/gyrA mutations can match combinations to local (and patient-level) resistance, with several trials and meta-analyses showing SGT outperforms short triple therapy and, in high-resistance settings, can rival or exceed empiric quadruple regimens when feasible (115–118). Second, biofilm-targeted adjuncts like N-acetylcysteine may improve antibiotic penetration. NAC is a mucolytic agent that can disrupt the extracellular polymeric matrix of *H. pylori* biofilms, thereby exposing bacteria that are otherwise protected from antimicrobial agents and host immune defenses. By destabilizing biofilm architecture, NAC may enhance antibiotic access to bacterial cells and improve eradication efficacy, particularly in persistent or refractory infections (119, 120). Although early clinical trials and meta-analyses have reported mixed results, this strategy remains of interest for patients with multiple treatment failures in whom standard antibiotic combinations are less effective (119, 120).

## Future directions

High-resolution human maps are redefining the infection of *H. pylori*. Combined single-cell RNA sequencing (scRNA-seq) and spatial transcriptomics of human stomach now resolve epithelial lineages, immune niches, and cytokine circuits at gland-level resolution, offering candidate biomarkers and therapeutic entry points (121). Recent single-cell studies focused on *H. pylori*–associated disease further chart cell-state transitions across gastritis-metaplasia-carcinoma, highlighting inflammatory fibroblasts, altered epithelial programs, and myeloid polarization that could be tracked longitudinally through eradication (122, 123). Moreover, a study combining metagenomic and scRNA-seq compared infected and uninfected human mucosa, adding the microbiome context needed to interpret these immune states *in situ*. Multi-omic patient profiling could guide individualized eradication strategies plus targeted immunomodulators, with tissue or stool biomarkers serving as early readouts of durable control (121, 124).

Organoid systems enable controlled testing of host-directed and microbiota-sparing interventions under near-physiologic conditions. A recent study introduced a homeostatic organoid-on-a-chip platforms that preserve apical acidity and support persistent *H. pylori* colonization, which can model *H. pylori* niche establishment and long-term colonization of the gastric epithelium (125). Using gastric organoid to further model the epithelial-microbe crosstalk will provide a practical roadmap for integrating immune cells to study barrier repair, adjuvants, and candidate immunotherapies before clinical translation (126, 127).

Given the critical role of MAIT cells in mucosal immunity, MAIT cell-based therapy for *H. pylori* infection represents a promising future direction. Human gastritis data implicate an OX40/OX40L axis that drives a highly activated MAIT phenotype producing IL-9, which correlated with mucosal inflammation (48). This finding raising testable ideas for selective costimulatory blockade in MAIT cell adoptive transfer therapy. Parallel advances in MR1 antigen handling, particularly efficient delivery of 5-OP-RU via soluble MR1 monomers, open experimental routes to visualize and perturb MAIT signaling directly within gastric tissues (128).

In short, the convergence of high-resolution human mapping, physiologic gastric models, and tractable MAIT/MR1 biology positions the field to move from population-level regimens toward immune-informed, tissue-targeted strategies that couple durable eradication with prevention of downstream gastric disease.

## Concluding remarks

*H. pylori* persists through a finely tuned interaction with the gastric mucosa, where epithelial defenses, myeloid polarization, and tissue-resident lymphocytes shape outcomes that range from asymptomatic carriage to ulceration and cancer. Within this ecosystem, MAIT cells illustrate the promise and peril of mucosal immunity: they respond rapidly to microbial metabolites yet can amplify pathology via an OX40/OX40L axis that drives IL-9–producing, highly activated states in gastritis (48, 124).

Clinically, the most immediate gains come from rigorous, resistance-aware care. The 2024 ACG guideline centers 14-day optimized bismuth quadruple therapy as default first-line when susceptibility is unknown, with vonoprazan-based regimens and rifabutin triple as context-dependent alternatives, and mandates test-of-cure after therapy (70). The Maastricht VI/Florence consensus complements this by emphasizing susceptibility-guided selection when feasible, an approach that can conserve antibiotic utility across diverse resistance ecologies (73).

Next-generation tools now offer the resolution and testbeds to turn mechanism into medicine. Single-cell and spatial maps of human stomach chart epithelial programs, myeloid states, and tissue-resident lymphocyte phenotypes across infection and premalignant change (121). Recent work has distilled spatial 26-gene signatures that localize to high-risk metaplastic glands and may serve as early biomarkers in eradication or prevention trials (129). Parallel metagenomic with single-cell atlases integrate the microbiome context of mucosal immunity (124). *In vitro*, homeostatic organoid-on-a-chip systems that preserve apical acidity and sustain persistent *H. pylori* colonization provide physiologic platforms to co-culture immune cells and to test antibiotics, acid suppression strategies, mucosal adjuvants, and MAIT/MR1-targeted interventions before clinical translation (125, 128). Taken together, integrating these tools with guideline-concordant antibiotics, selective immunomodulators, and vaccine development offers a practical path from population-level care to immune-informed, tissue-targeted control of *H. pylori* disease.
